# Enhancing grain boundary ionic conductivity in mixed ionic–electronic conductors

**DOI:** 10.1038/ncomms7824

**Published:** 2015-04-10

**Authors:** Ye Lin, Shumin Fang, Dong Su, Kyle S Brinkman, Fanglin Chen

**Affiliations:** 1 Department of Mechanical Engineering, University of South Carolina, Columbia, South Carolina 29208, USA; 2 Center for Functional Nanomaterials, Brookhaven National Laboratory, Upton, New York 11973, USA; 3 Department of Materials Science and Engineering, Clemson University, Clemson, South Carolina 29634, USA

## Abstract

Mixed ionic–electronic conductors are widely used in devices for energy conversion and storage. Grain boundaries in these materials have nanoscale spatial dimensions, which can generate substantial resistance to ionic transport due to dopant segregation. Here, we report the concept of targeted phase formation in a Ce_0.8_Gd_0.2_O_2−δ_–CoFe_2_O_4_ composite that serves to enhance the grain boundary ionic conductivity. Using transmission electron microscopy and spectroscopy approaches, we probe the grain boundary charge distribution and chemical environments altered by the phase reaction between the two constituents. The formation of an emergent phase successfully avoids segregation of the Gd dopant and depletion of oxygen vacancies at the Ce_0.8_Gd_0.2_O_2−δ_–Ce_0.8_Gd_0.2_O_2−δ_ grain boundary. This results in superior grain boundary ionic conductivity as demonstrated by the enhanced oxygen permeation flux. This work illustrates the control of mesoscale level transport properties in mixed ionic–electronic conductor composites through processing induced modifications of the grain boundary defect distribution.

## Abstract (web summary)

The performance of mixed ionic–electronic conductors used in energy conversion and storage devices can be restricted by their high grain boundary resistance. Here, the authors present the formation of an emergent phase to alleviate dopant segregation at the grain boundary and enhance ionic conductivity.

## Graphical abstract

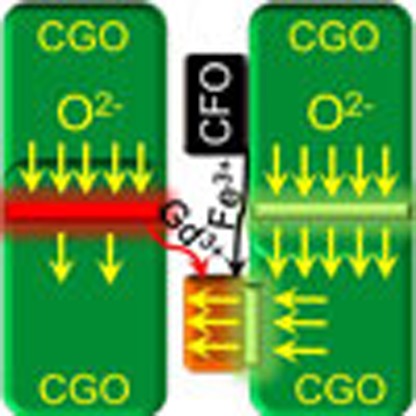

Mixed ionic–electronic conductors (MIECs) are widely used in semiconductors, electrochemical energy storage materials, electrodes of fuel cells and batteries, separation membranes and catalysts with various requirements for chemical, electrical, thermal and mechanical properties^1^,^2^,^3^,^4^,^5^,^6^,^7^,^8^,^9^,^10^,^11^. Single-phase MIECs seldom fulfill all of the requirements for these diverse applications. For example, typical perovskite-type MIECs exhibit structural instability in the extreme oxygen chemical potential gradients encountered in specific applications, including their use as anodes for solid oxide fuel cells and membrane reactors for partial oxidation of natural gas^12^. Composite MIEC materials consisting of separate ionic and electronic conductive phases offer a promising and robust material solution to this challenge. Property tuning appears to be flexible and straightforward in dual-phase MIECs (DP-MIECs), as both the ionic conductors and the electronic conductors have been well developed for many decades^13^,^14^,^15^,^16^. However, the performance of DP-MIECs is not necessarily controlled by the properties of individual constituents. Phase interactions and altered interfaces such as grain boundaries may play a key role in determining the overall device performance. Grain boundaries are believed to dominate the overall ionic conductivity through structural disorder, solute segregation, oxygen vacancy depletion and the formation of precipitates from extraneous phases^17^,^18^. In the vicinity of extended structural defects such as grain boundaries or edge dislocations, the concentration of each type of point defect is controlled by the difference in the standard chemical potential between the grain interior and the grain boundary/dislocation core^19^,^20^,^21^. A well-studied example is Ce_0.8_Gd_0.2_O_2−δ_ (CGO), which is an excellent choice for an oxygen ionic conductor due to its high oxygen ionic conductivity and structural stability. Doping with Gd^3+^ results in negatively charged gadolinium dopants on Ce^4+^ lattice sites (
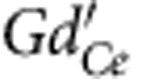
), which are charge compensated by positively charged oxygen vacancies (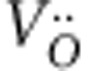). These defect pairs form defect associates due to Coulombic attraction, leading to an inhomogeneous distribution of charges and an inherent increase in the barrier for oxygen ion transport^22^,^23^,^24^,^25^. In particular, a positive space charge potential is observed in the grain boundary core of CGO, indicative of an accumulation of positively charged oxygen vacancies^26^. The characteristics of a space charge potential barrier extending from the grain interior to grain boundary core include an increase in the Gd/Ce atomic ratio and the Ce M_5_/M_4_ ratio, together with a decrease in the O/Ce atomic ratio. For example, the grain boundary core stoichiometry of Ce_0.8_Gd_0.2_O_2−δ_ has been reported to be Ce_0.59±0.04_Gd_0.41±0.04_O_1.24±0.17_ (ref. 26). This results in the depletion of oxygen vacancies in the space charge layer, and leads to a decrease in the grain boundary conductivity by several orders of magnitude as compared with the bulk conductivity in single-phase CGO^27^,^28^.

Compared with single-phase CGO, the ionic–electronic transport paths in CGO-based DP-MIECs should be more complex due to the emergence of new phases with additional grain boundary interfaces. In addition, the structure, composition and charge distribution in the grain boundaries can also change during fabrication of multiphase composites. Additional phases may be formed due to cation migration between the starting components at elevated temperature. New crystalline phases have the potential to either promote or obstruct ionic–electronic transport, depending on their structures and compositions^29^. The emergence of additional crystalline phases, therefore, provides an opportunity for modification of the inherent ionic transport barriers in the starting phases, such as the space charge-induced depletion of oxygen vacancies in the CGO–CGO grain boundary. However, the charge distribution at the CGO–CGO grain boundary has not yet been investigated in DP-MIECs. DP-MIECs based on CGO–AFe_2_O_4_ (A=Ni, Co or Mn) possess both high tolerance to CO_2_ and significant oxygen permeation performance, making them very attractive for post-combustion carbon capture and sequestration^30^,^31^,^32^,^33^,^34^, as well as anode for solid oxide fuel cells. Therefore, we have chosen CGO–CoFe_2_O_4_ (CFO) as a model system for general MIEC composite studies. We have investigated the influence of emergent crystalline phase formation on the defect structure at the grain boundary of the ionic conductive component CGO. Lessons learned from these investigations will result in significant benefit to related fields on the basis of ionic conduction processes for energy conversion and storage such as fuel cells and batteries.

Although the space charge layer effects have been observed for decades in single-phase CGO, it has never been completely controlled or modified due to the experimental difficulties associated with the small spatial size of the grain boundaries and oxygen ions (∼1–50 and 0.14 nm, respectively), together with the light weight of oxygen ions^20^,^27^,^35^. Microstructure and chemical composition analysis at the atomic scale relies on techniques such as scanning transmission electron microscopy (STEM) coupled with electron energy loss spectroscopy (EELS). STEM-EELS is capable of simultaneously mapping the atomic/electronic structure of light elements such as oxygen at adequate spatial resolution. Although the polycrystalline nature of the composites makes it difficult to obtain an atomic resolution image of both the CGO and CFO across the grain boundary, the electronic state of the elements across the boundary can be identified by STEM-EELS line scan crossing the grain boundary in steps of a few nanometers^36^.

In this study, we improve the oxygen ion transport by minimizing the space charge effect in the CGO–CGO grain boundaries through a controlled phase reaction between the CGO and CFO phases. A panorama of the atomic/electronic structure, relative atomic ratios and local dopant profiles near all types of grain boundaries in the MIEC composites is simultaneously probed and resolved by STEM-EELS in high spatial resolution (<1.3 Å) and energy resolution (<0.35 eV). The distribution, chemical composition and electronic fingerprint of an emergent Gd, Fe-rich phase formed during processing of the CGO–CFO composites are investigated by (S)TEM-energy dispersive X-ray spectrometry (EDX)/EELS and selected area electron diffraction (SAED). Finally, the oxygen permeation flux of these composites is measured to demonstrate their enhanced oxygen ionic conductivities.

## Results

### Morphology and phase structure of the composite MIECs

**Figure 1:**
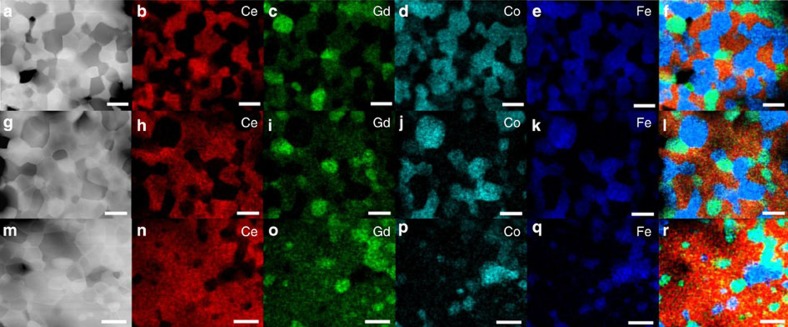
Phase identification by STEM-EDX. (**a**) High angle annular dark-field (HAADF) image showing the area of EDX mapping, (**b**–**e**) EDX element mapping in STEM mode for Ce, Gd, Co, Fe elements and (**f**) phase distinguishable map after combining the EDX signals of **b**–**e** for the CGO–CFO5050 sample; (**g**–**l**) and (**m**–**r**) are corresponding results for CGO–CFO6040 and CGO–CFO8020 samples, respectively. Scale bars, 1 μm.

Three composite MIECs were prepared using CGO and CFO with volume ratios of 50:50, 60:40 and 80:20 (denoted as CGO-CFO5050, CGO-CFO6040 and CGO-CFO8020, respectively). The compositions of the sintered composites were then examined by EDX mapping of Ce, Gd, Co and Fe in STEM mode. In addition to the two starting phases (CGO and CFO), the emergence of a new phase was observed. The three different phases are coloured in light green, orange and blue in the compositional images (Fig. 1f,l,r). The light green phase contains Gd, Fe, Co and Ce (denoted as GFCCO); the orange phase contains Ce and Gd (denoted as CGO); and the blue phase contains Co and Fe (denoted as CFO). High-angle annular dark-field (HAADF) images (Fig. 1a,g,m) in the STEM mode also revealed three phases from their contrasts (bright, grey and dark), corresponding to GFCCO, CGO and CFO phases with different atomic number (*Z*) contrast, respectively. The newly formed GFCCO phase is the product of a chemical reaction between CGO and CFO, as shown in equation (1) in which CGO–CFO6040 is chosen as an example:


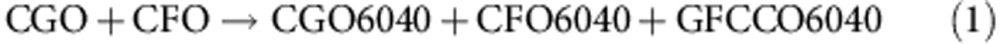


where CGO6040, CFO6040 and GFCCO6040 correspond to new CGO, CFO and GFCCO phases formed in the CGO–CFO6040 sample after sintering. The GFCCO phase is predominantly surrounded by the CGO phase rather than the CFO phase. In CGO–CFO8020 (Fig. 1r), many GFCCO particles are found inside the CGO at a great distance away from CFO particles, suggesting that the GFCCO phase is formed through the migration of Fe and Co, probably in the form of a liquid phase. CGO has a much higher sintering temperature than CFO (>1,400 °C and <1,100 °C, respectively)^37^,^38^. However, highly dense samples (relative density >95%) have been achieved for all the three CGO–CFO composites after sintering at only 1,300 °C, presumably because cobalt oxide leads to a liquid phase-mediated densification of ceria during the sintering process^39^. The Fe-, Co-containing liquid phase can easily transport the Fe and Co ions required for the reaction that forms the emergent GFCCO phase. A similar compositional investigation of single-phase CGO revealed that the Gd ion concentration at the CGO–CGO grain boundary core was much higher than the average concentration in the grain interior of single-phase CGO (∼40% compared with ∼20%, ref. 26). Therefore, the reaction that results in GFCCO phase formation is most likely initiated at the CGO–CGO grain boundary zone (reaction mechanism is suggested in Supplementary Fig. 1), resulting in the reduction of Gd ion concentration, which mitigates Gd accumulation in the CGO–CGO grain boundary. The origin of the space charge layer and poor grain boundary ionic conductivity in CGO is attributed to Gd accumulation, coupled with oxygen vacancy depletion in the grain boundary. Therefore, a change in Gd ion concentration in the CGO–CGO grain boundary can dramatically impact the oxygen ionic transport. To confirm this point, we carefully investigated the energy-loss near-edge structure (ELNES) of the element edges and the change of the extracted charge ratios near the grain boundary regions in all the composites.

### Grain boundary studies by STEM-EELS

**Figure 2:**
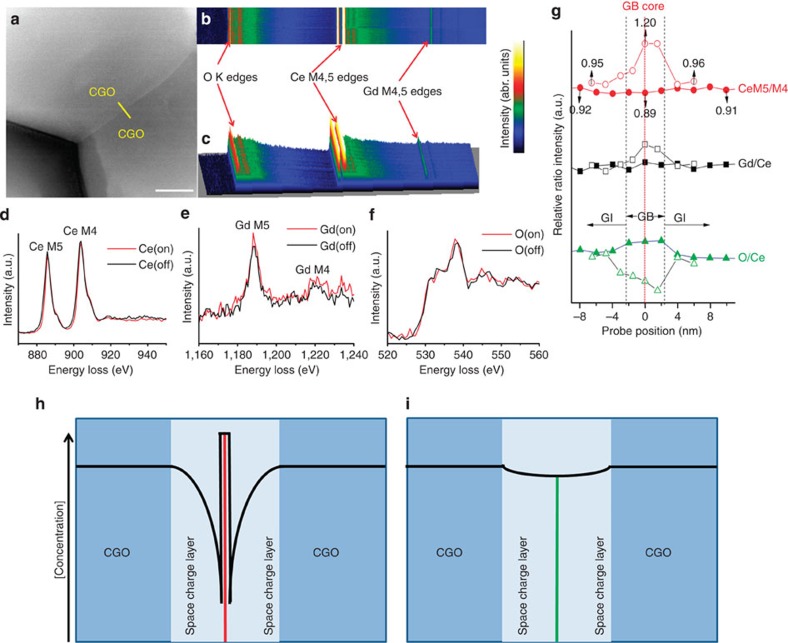
CGO–CGO grain boundaries in CGO–CFO6040 revealed by STEM-EELS. (**a**) The survey image including the EELS line scan across the CGO–CGO grain boundary (GB) from the CGO–CFO6040 composite; (**b**) and (**c**) are EELS line scan signal profiles presenting in two-dimensional and three-dimensional mode, respectively; (**d**), (**e**) and (**f**) are ELNES spectra of Ce M_4,5_, Gd M_4,5_ and O K edges extracted at (marked by on) or ∼10 nm away from (marked by off) the CGO–CGO grain boundary core, respectively; (**g**) profile of the CeM_5_/M_4_, Gd/Ce and O/Ce ratio near the CGO–CGO boundary (The solid symbol results are from this work, while the hollow symbol results are from single-phase CGO^26^. The grain boundary thickness of both samples is ∼4 nm); The proposed oxygen vacancy concentration profiles near the CGO–CGO grain boundary zone of (**h**) single-phase CGO and (**i**) CGO–CFO6040 composite. The GB core was highlighted by red and green line in **h** and **i**, respectively. Scale bar, 100 nm (**a**).

Figure 2 shows the compositional and charge distribution variations for the CGO–CGO grain boundary in the CGO–CFO6040 composite acquired by EELS line scan in the STEM mode. Figure 2g shows a significant difference in the Ce M_5_/M_4_, Gd/Ce and O/Ce ratios in the CGO–CGO grain boundary region in CGO–CFO6040 as compared with single-phase CGO^26^. The Gd/Ce ratio in the CGO–CGO grain boundary core of single-phase CGO is significantly higher than that in the grain interior, demonstrating the accumulation of Gd ions in the grain boundary and concomitant formation of a space charge layer^26^. The accumulation of Gd ions is closely correlated to the concentration of oxygen vacancies in the space charge layer, which is reflected by the ratio of Ce M_5_/M_4_. The Ce M_4,5_ and Gd M_4,5_ edges of the CGO–CGO grain boundary in single-phase CGO shifted to higher energy, and the Ce M_5_/M_4_ ratio increased 26.3% (0.95→1.20) at the grain boundary core compared with the grain interior^40^. In contrast, the ELNES spectra of Ce M_4,5_ edges, Gd M_4,5_ edges and O K edges at the CGO–CGO grain boundary core in the CGO–CFO6040 composite are all very similar to those from the CGO grain interior (∼10 nm away from core, Fig. 2d–f). There were no obvious edge position shifts or shape changes observed, indicating similar local chemical states, symmetry and atomic environment extending from the grain boundary to the grain interior for the CGO phase inside the CGO–CFO6040 composite^41^,^42^. Furthermore, the Ce M_5_/M_4_ only changed by 3.3% from the grain boundary core to the grain interior (0.92→0.89). This provides additional confirmation that the oxygen vacancy concentration at the CGO–CGO grain boundary core in CGO–CFO6040 is similar to the concentration in the CGO grain interior. Therefore, the depletion of oxygen vacancies inside the space charge layer, observed in single-phase CGO was significantly mitigated and a nearly constant distribution of oxygen vacancy concentration is expected along the CGO–CGO grain boundary inside the CGO–CFO6040 composite. A schematic is shown in Fig. 2h,i to conceptually understand the difference of the space charge effect between single-phase CGO and the modified CGO phase inside the CGO–CFO6040 composite. Figure 2h,i proposes that Gd ion accumulation and the resulting oxygen vacancy depletion in the CGO–CGO grain boundary are avoided in the CGO–CFO6040 composite through the *in situ* formation of a Gd- and Fe-rich GFCCO phase. Although similar effects were observed inside the CGO–CFO8020 composite, the CGO–CGO grain boundaries in CGO–CFO8020 were much thicker (∼42 nm compared with 4 nm in CGO–CFO6040, shown in Supplementary Fig. 2). The thicker grain boundary provides more resistance for the transport of oxygen ions^17^.

In addition to the interface of the primary oxygen ion conductor CGO–CGO, there are five additional types of grain boundaries inside these composites: CGO–GFCCO, CFO–GFCCO, GFCCO–GFCCO, CGO–CFO and CFO–CFO. The CGO–GFCCO grain boundary is especially important as it will determine whether the GFCCO phase will serve as a blocker or promoter for oxygen ion transport. Supplementary Figures 3 and 4 show the inter-diffusion of the elements and the change of the oxygen stoichiometry along the CGO–GFCCO boundaries from all of the composites studied in this work. The CGO–GFCCO boundary inside the CGO–CFO6040 composite displayed the shortest transport path (∼27 nm) and the least oxygen vacancy concentration fluctuation among the three composites. This resulted in the smallest resistance for oxygen ions transport among the CGO–GFCCO boundaries. On the basis of recent results using X-ray nanotomography, the volume ratio of GFCCO phases in both CGO–CFO6040 and CGO–CFO5050 is ∼10%, and the continuity of the GFCCO particles is zero^43^. Therefore, the GFCCO–GFCCO gain boundary is not a contiguous ion transport path. The STEM-EELS line scan results on the remaining grain boundaries are shown in Supplementary Figs 5–7. There were no Co and Fe concentration variations observed near the CFO–CFO grain boundaries, and the CFO–CFO boundaries are expected to serve as excellent electronic conduction pathways.

### Phase compositions and structures of CGO–CFO composites

The STEM-EDX quantification results for Gd, Fe, Co and Ce contents of all the three CGO–CFO composites are shown in Supplementary Tables 1–3. In all the cases, the ratio of Gd/Ce and Fe/Co in CGO and CFO is significantly lower than those in the CGO and CFO starting powders (0.25 and 2.0, respectively). Despite differences in composition, the CGO and CFO phases retained their original structures inside the three CGO–CFO composites (fluorite structure with space group Fm-3m (225) and spinel structure with space group Fd-3m (227), respectively), as shown in the X-ray diffraction and SAED patterns in Supplementary Figs 8–11. The STEM-EDX quantification results also revealed that the GFCCO phases were rich in Gd and Fe. Simultaneous growth of GdFeO_3_ (perovskite) and Gd_3_Fe_5_O_12_ (garnet) was observed in Gd_2_O_3_–Fe_2_O_3_ diffusion couples at 1,200–1,400 °C with activation energies of ∼400 and ∼550 kJ mol^−1^, respectively^44^. Among the potential GFCCO phases, GdFeO_3_ is thermodynamically more favoured to form than Gd_3_Fe_5_O_12_. In all the samples, the peaks of the newly formed GFCCO phase match well with that of GdFeO_3_ (Supplementary Fig. 8). However, the peaks of Gd_3_Fe_5_O_12_ cannot be distinguished due to significant overlap with the CGO, CFO and GdFeO_3_ diffraction peaks. Although X-ray diffraction patterns provide the best macroscopic structural information for phase identification, SAED patterns can provide more precise structural information of selected GFCCO particles (Supplementary Fig. 12). The results of SAED studies revealed that the GFCCO5050 and GFCCO8020 particles displayed a garnet A_3_B_5_O_12-δ_-like structure, whereas the GFCCO6040 particles possessed a perovskite-like structure. Higher oxygen ionic conductivities have been observed in perovskite-type phases as compared with garnet-type phases^45^.

Another pronounced feature of the GFCCO phase is its high *Z*-contrast. HAADF images (Fig. 1a,g,m) indicated that the average atomic number (*Z*_avg_) of the GCFFO phase was higher than the *Z*_avg_ for CGO. We have estimated that GFCCO phase has a high level of oxygen vacancies on the basis of the results of average atomic number (Supplementary Note 1). A high level of oxygen vacancies, together with the unique perovskite structure make the perovskite-type GFCCO a candidate for fast oxygen ionic conduction, providing additional paths for oxygen ion transport.

### Proposed models for ionic and electronic transport paths

**Figure 3:**
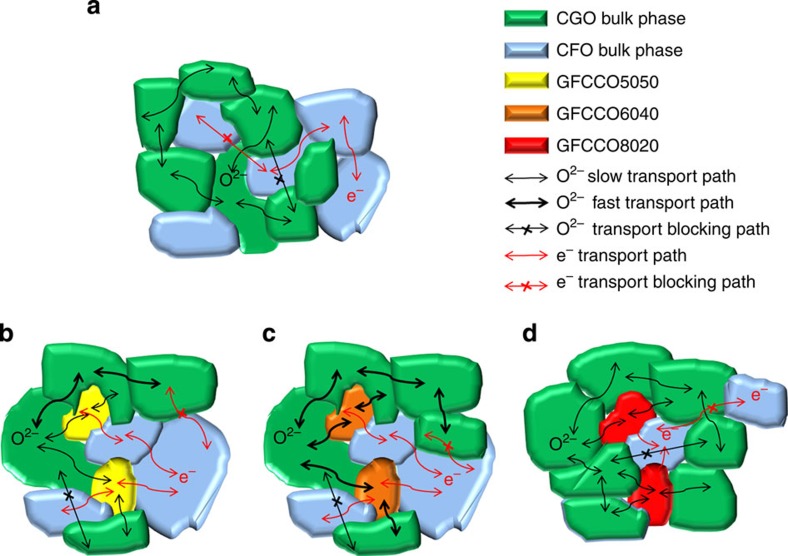
Proposed oxygen ionic and electronic transport paths. (**a**) Traditional dual phase MIECs (DP-MIECs) without formation of the third phase. (**b**–**d**) Novel ternary phase MIECs (TP-MIECs) shown as (**b**) CGO–CFO5050 (**c**) CGO–CFO6040 and (**d**) CGO–CFO8020 in this work.

Figure 3a shows the potential paths for oxygen ionic and electronic transport in DP-MIECs consisting solely of one ionic and one electronic conductive phase. The effective oxygen ionic conduction in DP-MIECs is limited by the space charge effect in the grain boundaries of the oxygen ionic conductor (such as CGO) and the increased tortuosity introduced by the electronic conductive phase. In the case of CGO–CFO composites, both the grain boundary conductivity and tortuosity are changed by the formation of an emergent GFCCO phase. The results of our microscopy and EELS studies can be summarized as follows: (a) the CGO–CGO grain boundaries in the CGO–CFO6040 composite conduct oxygen ions better than traditional CGO; (b) the GFCCO phase in the CGO–CFO6040 composite possesses a perovskite-like structure and high level of oxygen vacancies, providing additional pathways for oxygen ionic conduction; and (c) the CGO–GFCCO grain boundaries in the CGO–CFO6040 composite conduct oxygen ions better than those in CGO–CFO5050 and CGO–CFO8020 (Supplementary Fig. 3). On the basis of the results above, the oxygen ionic/electronic transport paths for all the composites are shown in Fig. 3b–d. In general, the CGO–CGO and CGO–GFCCO grain boundaries, along with the GFCCO6040 and CGO grains in CGO–CFO6040 are fast oxygen-ionic transport paths. The CGO–CFO5050 and CGO–CFO8020 possessed grain boundaries (CGO–GFCCO) and garnet-type GFCCO grains, which are expected to be slow oxygen-ionic transport paths. The electronic transport in CGO–CFO8020 is also limited because the volume ratio of CFO (20%) is below the percolation limit. Considering that the oxygen permeation process involves both oxygen ionic and electronic transportation, the oxygen permeation flux should increase in the following order: CGO–CFO6040>CGO–CFO5050> CGO–CFO8020, which was verified by oxygen permeation measurements.

### Oxygen permeation performance of CGO–CFO composites

**Figure 4:**
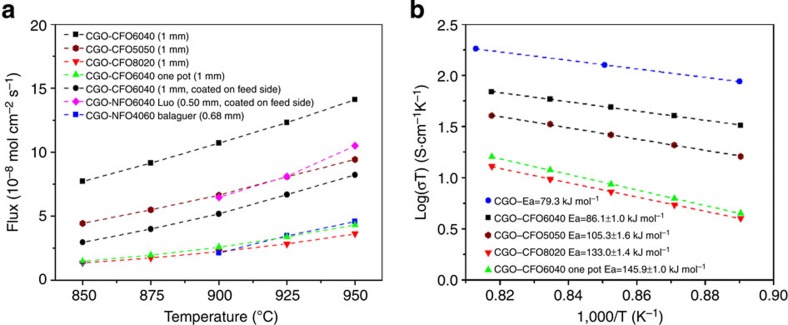
Oxygen permeation performance of the CGO–CFO composite MIECs. (**a**) Oxygen permeation flux of the CGO–CFO composites with different volume ratios measured between 850 and 950 °C. Feed gas: 150 ml min^−1^ air. Sweep gas: 30 ml min^−1^ helium. (**b**) The Arrhenius plot of the calculated ambipolar conductivities of CGO–CFO composites based on oxygen permeation flux. The ionic conductivity of single-phase CGO is shown for comparison.

Figure 4a shows the oxygen permeation flux of different CGO–CFO composite membranes, along with literature data for comparison. The flux of the membranes follows the predicted order based on oxygen ion and electron transport pathways: CGO–CFO6040>CGO–CFO5050>CGO–CFO8020. It should be noted that the CGO–CFO6040 shows a significantly higher flux than previously reported membranes based on the materials system CGO–AFe_2_O_4_. For example, a 1.0-mm-thick CGO–CFO6040 and 0.5-mm-thick Ce_0.9_Gd_0.1_O_2−δ_–NiFe_2_O_4_ (volume ratio 60:40, CGO–NFO6040) membranes displayed oxygen permeation flux values of 5.18 × 10^−8^ and 6.47 × 10^−8^ mol cm^−2^ s^−1^, respectively at 900 °C, with the presence of a CGO–Sm_0.5_Sr_0.5_CoO_3-δ_ (SSC) catalyst coated on the feed side^32^. The much thicker CGO–CFO6040 membrane examined in this work shows similar flux values compared with the CGO–NFO6040 composite. A CGO–CFO7525 sample prepared by the one-pot method exhibited very low flux (∼1.0 × 10^−8^ mol cm^−2^ s^−1^) at 900 °C (ref. 30), which may have been due to differences in the volume ratio of ionic-to-electronic conductive phases and surface exchange limitations due to the lack of a catalyst on the membrane surface. To clarify this, we prepared a CGO–CFO6040 sample by the one-pot method and measured the oxygen flux under the same conditions of a CGO–CFO6040 sample prepared by the powder-mixing method. Most CGO-based DP-composite membranes show higher performance when the grain sizes are smaller. For example, when CGO–Fe_2_O_3_ membranes prepared by the one-pot method were sintered at different temperatures to produce different grain sizes, the membrane with finer CGO and Fe_2_O_3_ grains showed higher oxygen flux^46^. The CGO–NiFe_2_O_4_ membrane prepared by the one-pot method also showed higher performance than samples prepared by the powder-mixing method. This is because the former contains finer CGO and NiFe_2_O_4_ grains and less homoaggregation of grains of the same phase than the latter^32^. One would expect CGO–CFO6040 prepared by the one-pot method to show a higher flux than samples prepared by the powder-mixing method. However, the flux of the CGO–CFO6040 samples prepared by the powder-mixing method was approximately four times higher than those prepared by the one-pot method at 900 °C (Fig. 4a). The total electrical conductivity of the CGO–CFO6040 samples was also several times higher than those of various CGO–CFO composites prepared by the one-pot method measured at 300 °C (shown in Supplementary Fig. 13, Supplementary Table 4). These major, unexpected differences are apparently not due to the difference in CGO, CFO grain sizes/distribution, but are closely related to the difference in the CGO–CGO grain boundaries and the formation of GFCCO phases. In CGO–Fe_2_O_3_, CGO–NiFe_2_O_4_, and CGO–CFO6040 one-pot membranes, no apparent phase reactions or new type of grains were found. Instead, a rim structure consisting of CGO and CFO nanoparticles was found at the grain boundaries in the CGO–CFO composites made by the one-pot method^30^. Therefore, the interfacial structure of CGO–CGO grain boundaries in these CGO-based composites will always hinder the oxygen ion transport, leading to high activation energies for oxygen permeation (135.3, 128±4 and 123.4±0.6 kJ mol^−1^ for CGO–Fe_2_O_3_, CGO–NiFe_2_O_4_ and CGO–CFO6040 one pot, respectively) and relatively low flux^32^,^46^. In contrast, the space charge effect near the grain boundary region was mitigated in the CGO–CFO composites prepared by the powder-mixing method by the formation of oxygen ionic conducting GFCCO phases. The activation energies calculated from the Arrhenius plot of flux and temperature are 68.8±1.0, 86.4±1.5 and 113.0±0.9 kJ mol^−1^ for CGO–CFO6040, CGO–CFO5050, CGO–CFO8020 prepared by the powder-mixing method, respectively. This further highlights the importance of the high CGO–CGO grain boundary conductivity in CGO–CFO6040 samples prepared by the powder-mixing method.

The flux ratio at 900 °C is 3.0:4.8:1.0 for the CGO–CFO5050, CGO–CFO6040 and CGO–CFO8020, respectively. The oxygen permeation flux of the CGO–CFO6040 membrane is ∼61% higher than the flux of the CGO–CFO5050 membrane at 900 °C. This is unexpected based solely on a 10% difference in the volume ratio of the oxygen ion conducting CGO phase. It was demonstrated that the CGO and CFO phases maintained the fluorite and spinel structures and exhibited similar Gd/Ce and Fe/Co ratios in these two composites (Supplementary Figs 9 and 10, Supplementary Tables 1 and 2). Therefore, the large difference in oxygen permeation flux may be closely related to the different structure of the GFCCO phases and CGO–GFCCO grain boundaries. The perovskite-type GFCCO6040 phase in CGO–CFO6040 has a structure permitting greater oxygen transport compared with the garnet-type GFCCO5050 in the CGO–CFO5050 (ref. 45). Moreover, the change in the oxygen vacancy concentration across the CGO–GFCCO grain boundary in the CGO–CFO6040 is much smoother than variations in the CGO–CFO5050 (Supplementary Fig. 3), which facilitates the transport of oxygen ions. The high level of oxygen flux in the CGO–CFO6040 sample is attributed to the cooperative effect of these factors. The poor performance of the CGO–CFO8020 composite is mainly attributed to the lack of electronic transport paths on the basis of percolation theory (Fig. 3d). The formation of nano-domains with oxygen vacancy associates (Supplementary Fig. 11c,f) inside the CGO phase in CGO–CFO8020 could also be a barrier for the oxygen ionic transportation^47^.

The ambipolar conductivities of the CGO–CFO composites were calculated (details shown in Supplementary Note 2, Supplementary Fig. 14) on the basis of the Wagner equation and are shown in Fig. 4b (ref. 48). The ambipolar conductivities of CGO–CFO6040 are 0.057, 0.042 and 0.029 S cm^−1^ at 950, 900 and 850 °C. As the electronic conductivity of CFO is >1 S cm^−1^ in this temperature region, the ambipolar conductivity can be estimated as the ionic conductivity^12^,^43^. After correcting for the CGO volume fraction in CGO–CFO6040 (57%, as determined by X-ray nanotomography^43^), the ionic conductivities are 0.10, 0.073 and 0.051 S cm^−1^ at 950, 900 and 850 °C, respectively. These values are very close to the ionic conductivity of CGO (0.14, 0.11 and 0.078 S cm^−1^ at 950, 900 and 850 °C, respectively) measured by the DC four-probe method and corrected by the ionic transference number^49^. The ionic conductivities of CGO–La_0.8_Sr_0.2_Fe_0.8_Co_0.2_O_3−δ_ and CGO–La_0.7_Sr_0.3_MnO_3−δ_, in contrast, are about one order of magnitude lower than that of CGO^29^. Furthermore, the activation energies for ionic conductivity are 79.3±0.0, 86.1±1.0, 105.3±1.6 and 133.0±1.4 kJ mol^−1^ for single-phase CGO, CGO–CFO6040, CGO–CFO5050 and CGO–CFO8020, respectively, as plotted from Fig. 4b. The activation energy of CGO–CFO6040 is very close to that of single-phase CGO in the similar temperature range. In comparison, the activation energies for ionic conductivity of conventional DP-MIECs based on doped ceria are much higher (108±14, 106±7 and 154±10 kJ mol^−1^ for CGO–La_0.8_Sr_0.2_Fe_0.8_Co_0.2_O_3−δ_, CGO–La_0.7_Sr_0.3_MnO_3−δ_, and Ce_0.8_Sm_0.2_O_2−δ_–La_0.8_Sr_0.2_CrO_3−δ_, respectively)^29^,^50^. The high ionic conductivity and the low activation energy of the CGO–CFO6040 composite suggest the effectiveness of fast oxygen-ionic transport paths through the GFCCO6040 phase and the CGO–CGO, plus the CGO–GFCCO grain boundaries.

## Discussion

We correlated the performance of CGO–CFO composite MIECs with the charge distribution near the CGO–CGO grain boundaries and the structure of the newly formed GFCCO phase using spatially resolved EDX/EELS, HADDF-STEM imaging and SAED. Negligible differences in Gd/Ce, Ce M_5_/M_4_ and O/Ce ratios were found near the CGO–CGO grain boundary region in the CGO–CFO composites. The homogeneous charge distribution and uniform chemical environment along the grain boundary enable fast oxygen ionic transport. In comparison, the accumulation of Gd ions and depletion of oxygen vacancies at the CGO–CGO grain boundary in single-phase CGO deteriorate its grain boundary conductivity. Our findings show that this difference originates from cation migration driven by the formation of Gd- and Fe-rich GFCCO phases. The reaction most likely initiates at the CGO–CGO grain boundaries where Gd ions are known to accumulate. The reaction forming the GFCCO phase reduces the Gd content in the grain boundaries. The GFCCO6040 phase possesses a perovskite structure and high level of oxygen vacancies, which are characteristic of fast oxygen ion conductors. Furthermore, the CGO–GFCCO grain boundaries inside CGO–CFO6040 show nearly constant local oxygen stoichiometry, which also facilitates oxygen transport. The synergistic effect of fast oxygen transport in CGO–CGO, CGO–GFCCO grain boundaries, and the GFCCO6040 phase in CGO–CFO6040 results in the highest oxygen flux observed in the CGO–AFe_2_O_4_ materials system. These findings point out a novel method to optimize the performance of composite MIECs: an *in situ* phase reaction, which simultaneously reduces the space charge effect at the grain boundaries and creates new oxygen ionic conducting pathways. Engineering interfaces such as grain boundaries through an *in situ* phase reaction provides a promising path toward the design of high-performance materials for energy conversion and storage. This concept of intentionally targeting emergent phase formation, involving elements known to segregate at grain boundaries provides a new tool for materials system design. This concept was demonstrated for composite MIEC systems, but the approach can also be applicable to studies involving sintering aids in ionic conductors used as solid oxide electrolytes for a variety of energy conversion and storage systems. One example may be BaZr_0.8_Y_0.2_O_3−δ_, which possesses very high bulk proton conductivity but poor grain boundary proton conductivity^18^,^51^,^52^.

## Methods

### Sample preparation

CGO and CFO powders (12–50 nm and 20–50 nm respectively, based on TEM characterization, InfraMat Advanced Materials LLC, USA) with volume ratios of 50:50, 60:40 and 80:20 were ball-milled in ethanol for 20 h and then dried to obtain CGO–CFO composite powders. These powders were pressed into pellets in a 20-mm stainless steel die. The pellets were sintered in air at 1,300 °C for 2 h. CGO–CFO6040 was also prepared by a one-pot combustion method using metal nitrates and citric acid. The relative densities of the sintered composites measured by the Archimedes' method were all >95%.

### Electron microscopic and spectroscopic characterizations

Due to the polycrystalline nature of both CGO and CFO in the composites, a large thin area of the samples was needed for the TEM and STEM-EELS studies to obtain averaged information. Thin areas with a thickness ∼50 nm were obtained for all the three composites from the classic dimpling and ion milling method. The sintered CGO–CFO composites were first cut by a diamond saw and then by an ultrasonic disk cutter (Model 170) to produce disks of 3 mm in diameter. The thickness of the samples was then reduced to 150 μm by polishing using sandpaper, and then reduced to 30 μm by dimpling using a dimpler (Gatan 626). The final sample thickness of ∼50 nm was achieved by ion milling using an ion milling system (Fischione Model 1010). The same voltage, current and milling angel programs were applied for all the samples to keep the sample preparation consistent. No surface damage and obvious amorphous phases were observed with the final low energy ion milling processes for all of the three CGO–CFO samples. The powder samples for the TEM test were first prepared by supersonic dispersion of the powders in ethanol, dipping the powder suspension onto a 400 mesh Cu grid (Ted Pella, Inc.), followed by drying in air. HRTEM images and SAED patterns were acquired by using the JEOL 2100F TEM equipped with a Schottky field-emission gun (FEG) with Cs=1.0 mm operated at 200 kV. EDX mapping tests in the TEM mode were performed before taking HRTEM images and SAED to distinguish the different types of grains/grain boundaries from the composites. For each phase from different composites, local atomic composition and electron-ELNES for different edges both for the grain boundary and grain interior were characterized using a Cs-corrected Hitachi HD-2700C STEM equipped with a Cold-FEG gun, operated at 200 kV coupled with EELS. Images were acquired using a probe convergence semi-angle of 27 mrad, and the inner collection angle of the ADF detector was 53 mrad. The EELS data were collected using an Engina ER (Gatan) detector, with a collection half-angle of 26 mrad. STEM-EDX mapping was also taken by a 5030 EDX detector from Bruker, attached with the Hitachi STEM. The energy resolution is ∼1.3 eV at the dispersion of 1.25 eV per channel. The probe size was 1.3 Å, the beam current was 30∼40 pA, and the dwelling time was 3 s. Steps of 3∼5 Å were carefully selected to avoid the possible effects induced by the electron-beam damage. The signals of O K-edge, Gd M_4,5_-edges, Ce M_4,5_-edges, Fe L_2,3_-edges and Co L_2,3_-edges were obtained simultaneously and performed using a non-linear least squares fitting integrated with Digital Micrograph software.

### Oxygen permeation flux measurement

The sintered CGO–CFO membranes were polished to a thickness of 1.0 mm using sandpapers with different grits from P120 to P600. CGO–Sm_0.5_Sr_0.5_CoO_3-δ_ (SSC) catalyst layers with thickness of ∼20 μm were painted on both surfaces of the membranes to accelerate the oxygen exchange process on the surfaces. After drying, the membranes were directly sealed to an alumina tube using a glass ring (Schott 8252) at 1,000 °C for 2 h. The catalyst layers were *in situ* calcined in the sealing process. The oxygen permeation flux was measured between 850 and 950 °C. The feed gas was 150 ml min^−1^ air, while the sweep gas was 30 ml min^−1^ He. The sweep gas from the outlet was analysed by gas chromatography (Agilent 7890A). The leakage due to incomplete sealing was <1% and corrected before the calculation of oxygen flux.

### Other characterization

For the impedance tests, both surfaces of the sintered CGO–CFO pellets were polished using sandpaper with P2500 grit and painted with Pt paste. To ensure good contact between Pt electrodes and the samples, the pellets were sintered in air at 1,000 °C for 5 h with a heating and cooling speed of 5 °C min^−1^. The impedance tests were performed in air between 150 and 800 °C, using an IM6 electrochemical workstation (Zahner) in the frequency range of 10^−2^–10^6^ Hz. The fittings of the obtained impedance spectra with the corresponding equivalent circuit model were done using the software Z-View.

## Author contributions

Y.L. conceived and designed the experiments with assistance from S.F. and D.S. K.S.B. prepared the MIECs powder and composites. Y.L. and D.S. prepared the TEM samples and performed the microscopy tests and analysis. S.F. and Y.L. carried out the oxygen permeation experiments and X-ray diffraction test. Y.L. made the conductivity test. F.C. and K.S.B. conceived and supervised the project. Y.L. and S.F. composed the manuscript and prepared the figures. All the authors discussed the results, commented and revised the manuscript.

## Additional information

**How to cite this article:** Lin, Y. *et al*. Enhancing grain boundary ionic conductivity in mixed ionic–electronic conductors. *Nat. Commun.* 6:6824 doi: 10.1038/ncomms7824 (2015).

## Supplementary Material

Supplementary Information Supplementary Figures 1-14, Supplementary Tables 1-4, Supplementary Notes 1-2 and Supplementary References
