# Supplementary material for: Enhancing grain boundary ionic conductivity in mixed ionic–electronic conductors
Source: Nat Commun. 2015 Apr 10;6:6824. doi: 10.1038/ncomms7824 (PMC4403342; doi:10.1038/ncomms7824)
Supplement: Supplementary Information — Supplementary Figures 1-14, Supplementary Tables 1-4, Supplementary Notes 1-2 and Supplementary References [file ncomms7824-s1.pdf]

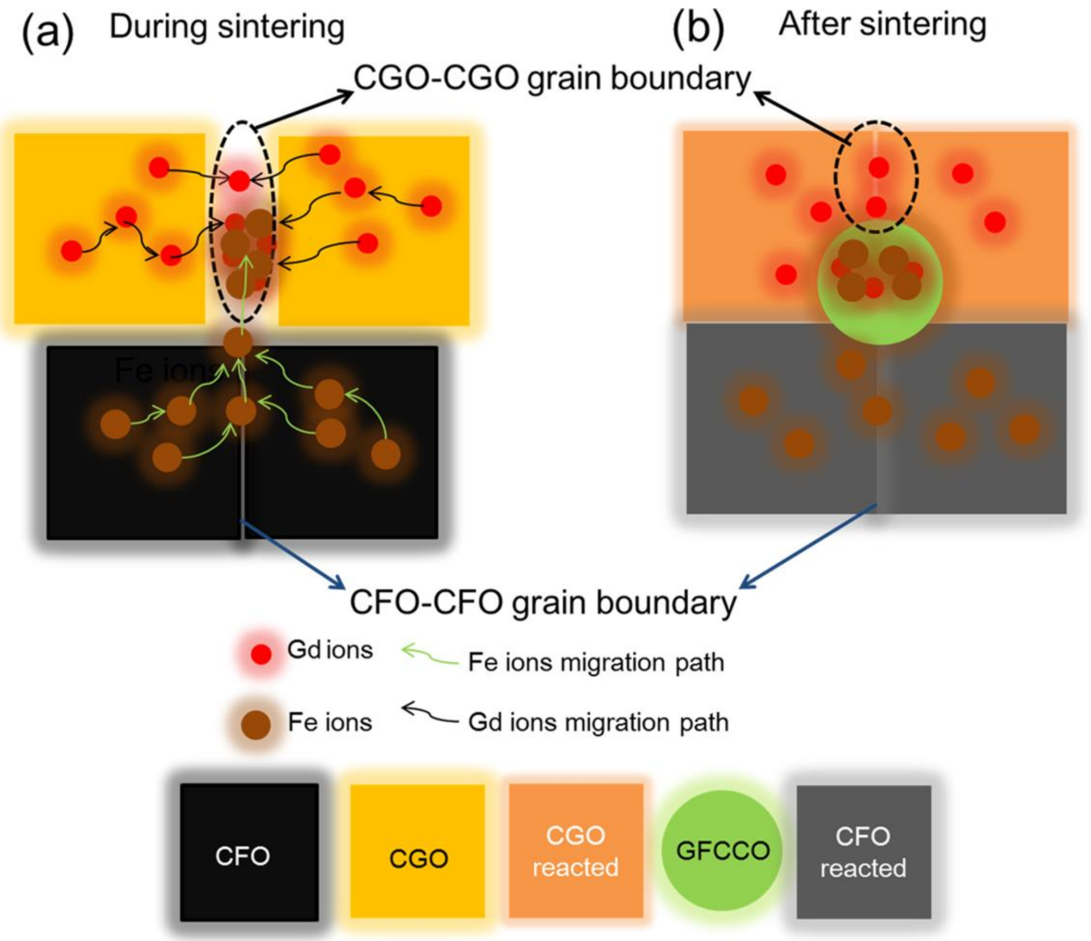

Supplementary Figure 1. Schematic of the proposed phase reaction mechanism between CGO and CFO. (a) Dopants (mainly Gd and Fe ions) moved inside the CGO-CFO composites during the sintering process, (b) the final state of the CGO-CFO composites.

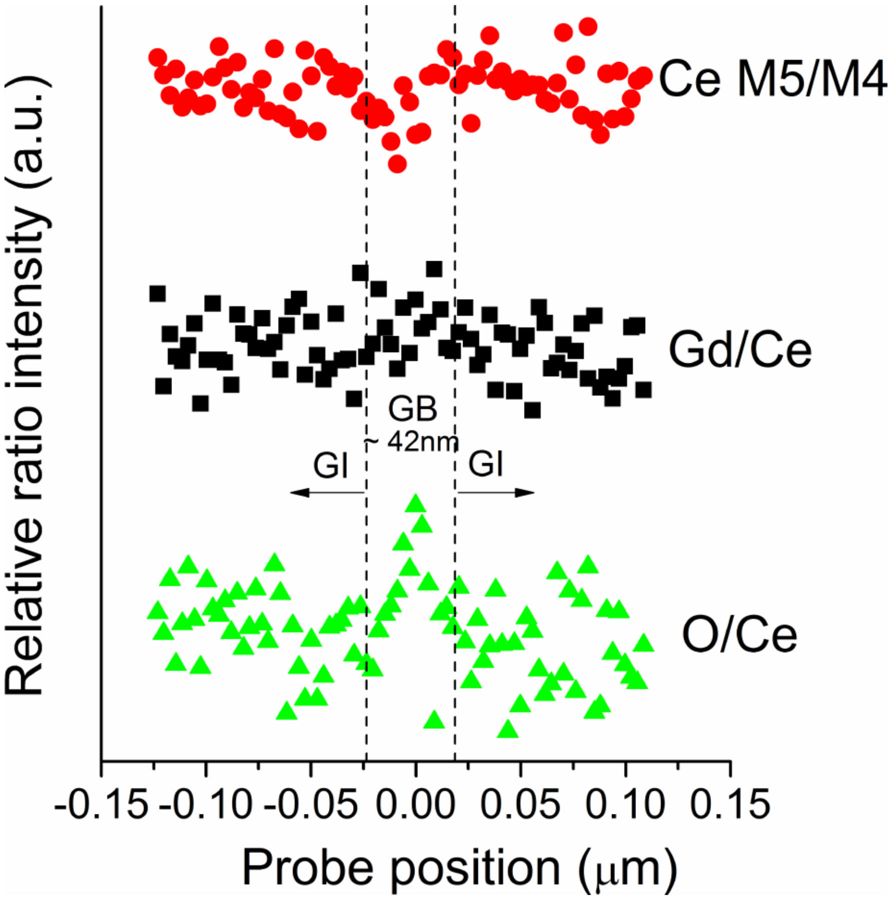

Supplementary Figure 2. The Ce M_5/M_4, Gd/Ce, and O/Ce ratio changes observed by EELS line scan near the CGO-CGO grain boundary in CGO-CFO8020. A long grain boundary (GB) thickness of ~42 nm is observed.

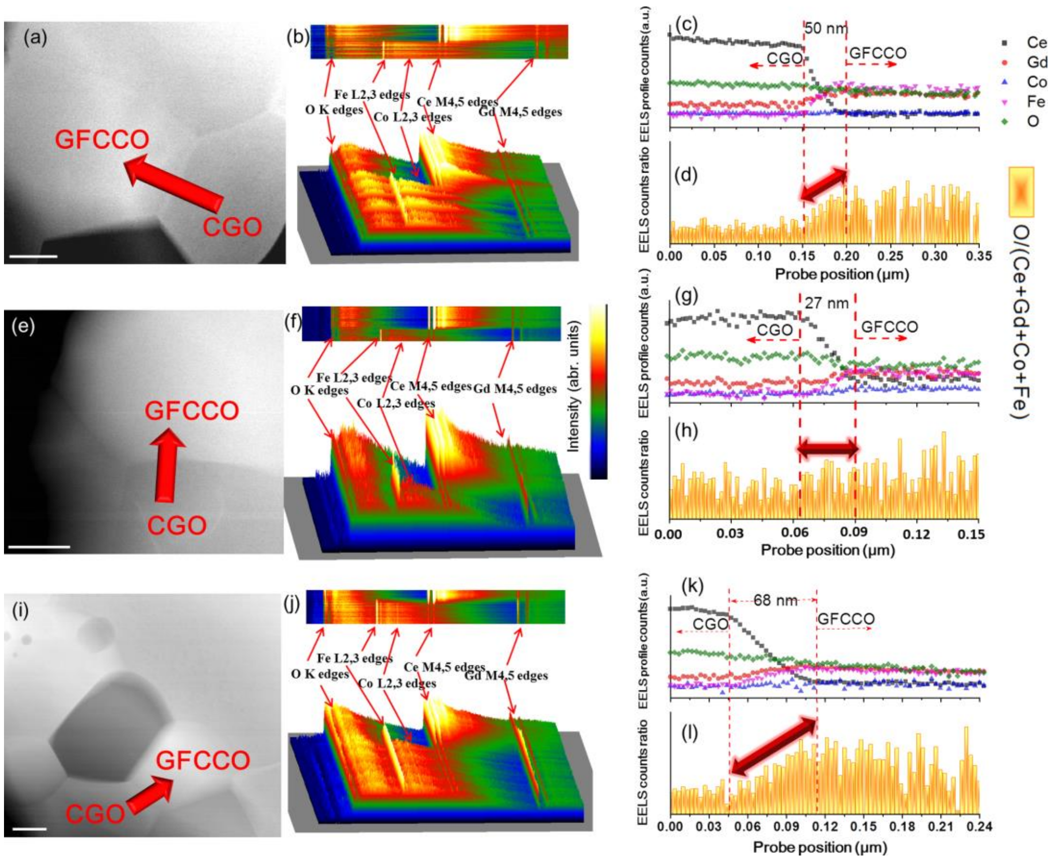

Supplementary Figure 3. CGO-GFCCO grain boundaries in CGO-CFO composites revealed by STEM-EELS. (a) The survey image including the EELS line scan position interfaces, (b) EELS line scan signal profiles presented in 2D&3D modes, (c) Extracted EELS signals for Ce, Gd, Co, Fe, and O element inter diffusion, and (d) Oxygen stoichiometry changes across the CGO-GFCCO for CGO-CFO5050 sample; (e-h) and (i-l) are similar results for CGO-CFO6040 and CGO-CFO8020, respectively. The grain boundary zone is indicated by dashed line. The thicknesses of the CGO-GFCCO boundaries are 50, 27, and 68 nm for CGO-CFO5050, CGO-CFO6040, and CGO-CFO8020, respectively. The signals of Gd, Fe, and Co increase and the Ce and O decrease across the CGO-GFCCO boundaries in all the three composites. All the CGO-GFCCO grain boundaries show high content of Ce. The oxygen stoichiometry represented by O/(Ce+Gd+Co+Fe) shows a platform for the CGO-GFCCO grain boundary in CGO-CFO6040 composite, while an obvious oxygen stoichiometry gradient is observed for those boundaries from the CGO-CFO5050 and CGO-CFO8020 composites. Scale bar is 0.2 μm.

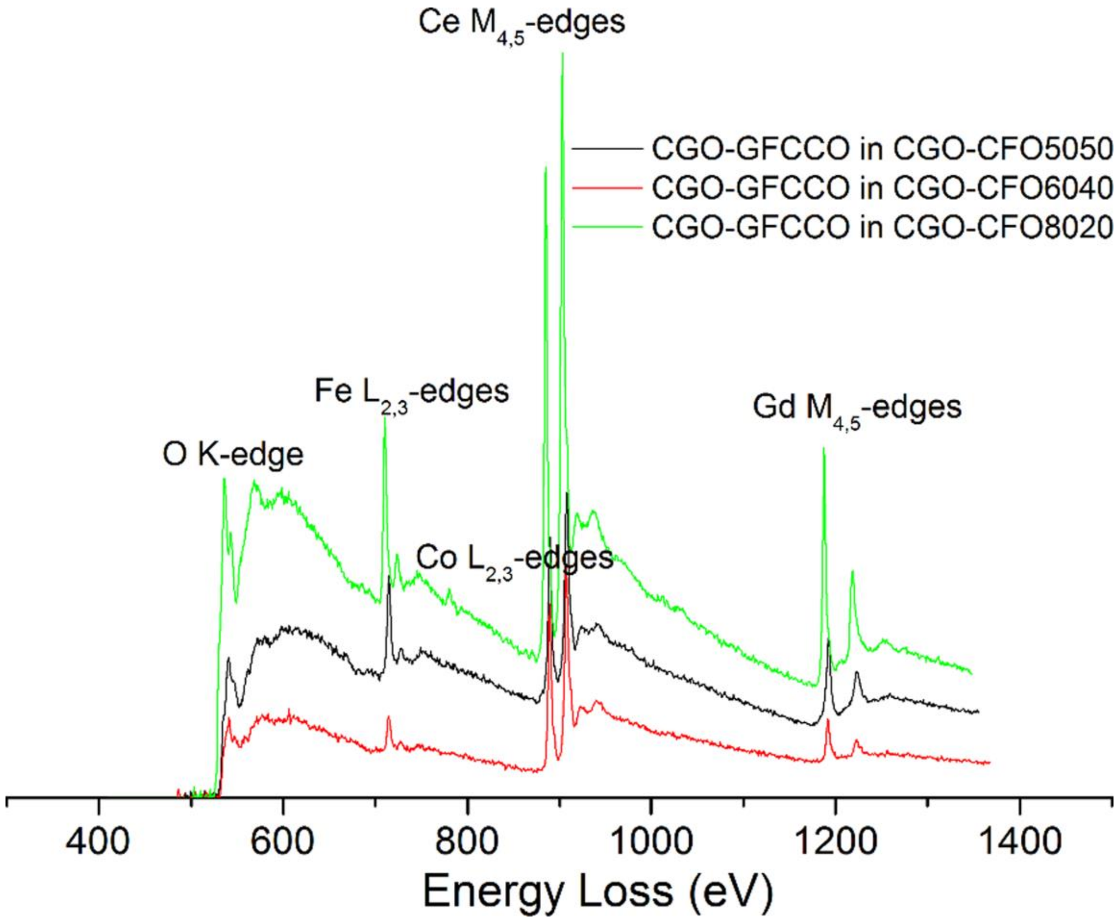

Supplementary Figure 4. Representative EELS spectra from the CGO-GFCCO grain boundary region inside different CGO-CFO composites. Similar ELNES edges are observed. The position/shape of the O K edge is retained across the CGO-GFCCO zone from all three composites.

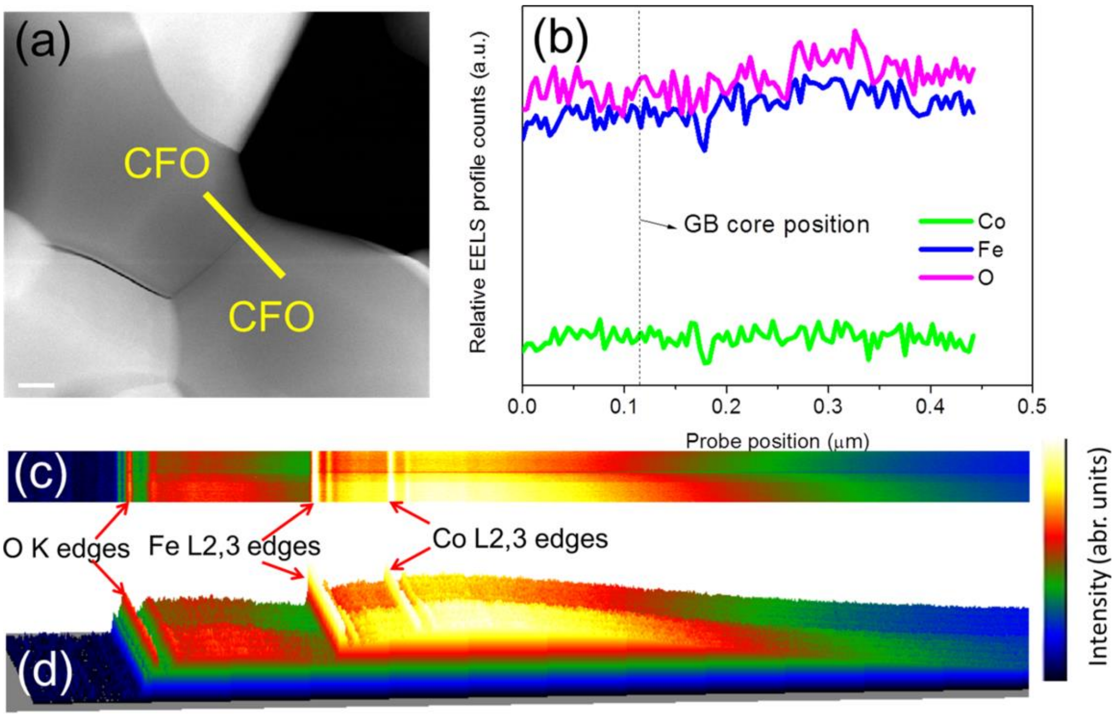

Supplementary Figure 5. (a) The survey image including the EELS line scan position across the CFO-CFO grain boundary in CGO-CFO6040; (b) EELS signal profiles during the line scan (the grain boundary core is indicated in dash line); (c)&(d) EELS line scan signal profiles presented in 2D&3D modes, respectively. After sintering, only Co, Fe, and O are observed both inside the CFO grains and in the CFO-CFO grain boundary region (c, d). Co and Fe in CFO still maintain a homogenous distribution both in the grain interior and in the CFO-CFO grain boundaries (b). Furthermore, unlike other grain boundaries, the thickness of the CFO-CFO grain boundary is non-distinguishable, as there is no variation in Co or Fe concentration near the CFO-CFO grain boundaries. Based on these findings, the CFO grains and CFO-CFO grain boundaries after the phase reaction are expected to function well as electronic transportation pathways. Scale bar is 200 nm.

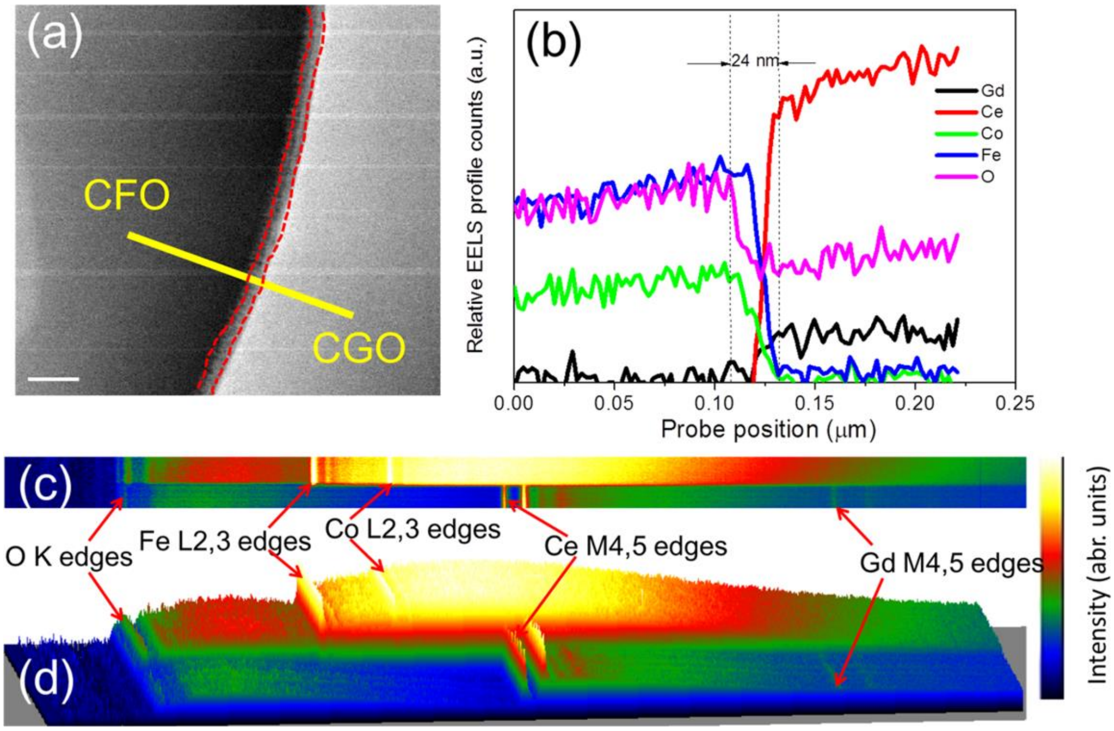

Supplementary Figure 6. (a) The survey image including the EELS line scan position across the CFO-CGO interface in CGO-CFO6040; (b) EELS signal profiles during the line scan (the grain boundary core are indicated by the dash line); (c)&(d) EELS line scan signal profiles presenting in 2D&3D modes, respectively. The thickness of CFO-CGO grain boundary is about 24 nm. The signals of Co, Fe, and O decrease while the Gd, Ce signals increase gradually from the CFO to the CGO phase. The rim structure near the grain boundary region as plotted by red dash line (a) showed clear Z-contrast. The rim structure near the CFO-CGO grain boundary was also found in the one pot derived CGO-CFO composites, and only nano-sized CGO and CFO grains but no $GdFeO_3$-related phases were found in the rim structure[1]. Scale bar is 50 nm.

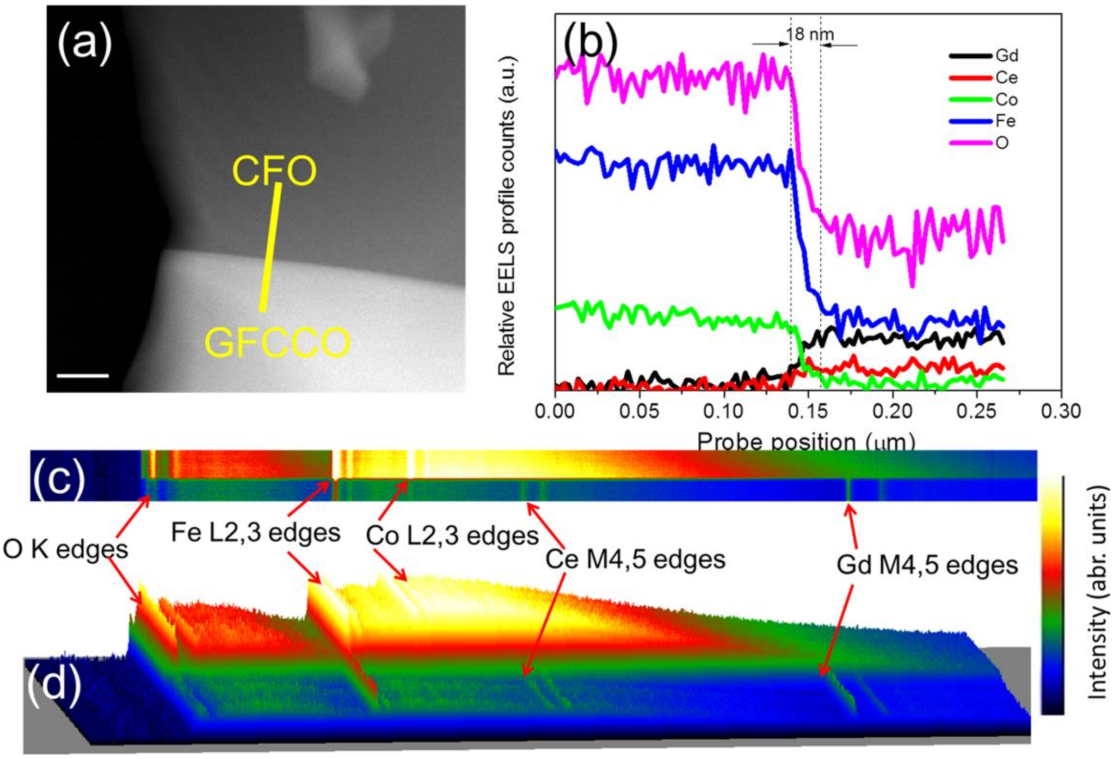

Supplementary Figure 7. (a) The survey image including the EELS line scan position across the GFCCO-CFO grain boundary in CGO-CFO6040; (b) EELS signal profiles during the line scan (The grain boundary core is indicated by the dash line); (c)&(d) EELS line scan signal profiles presented in 2D&3D modes, respectively. Scale bar is 100 nm.

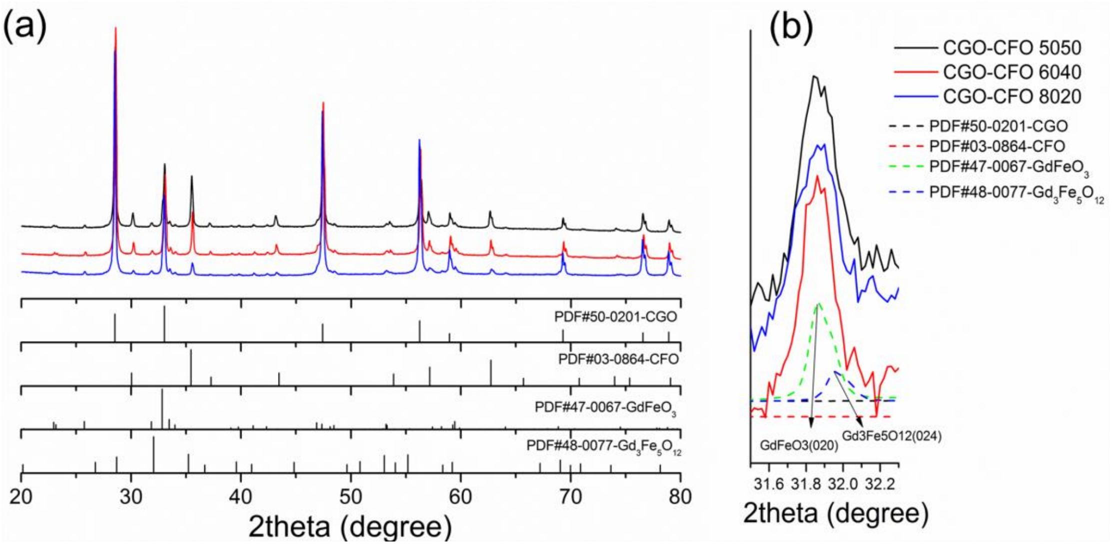

Supplementary Figure 8. (a) XRD patterns of sintered CGO-CFO5050, CGO-CFO6040, and CGO-CFO8020 composites and standard XRD patterns from CGO, CFO, $GdFeO_3$, and $Gd_3Fe_5O_{12}$ phases, (b) XRD patterns selected region with simulated standard CGO, CFO, $GdFeO_3$, and $Gd_3Fe_5O_{12}$ peaks.

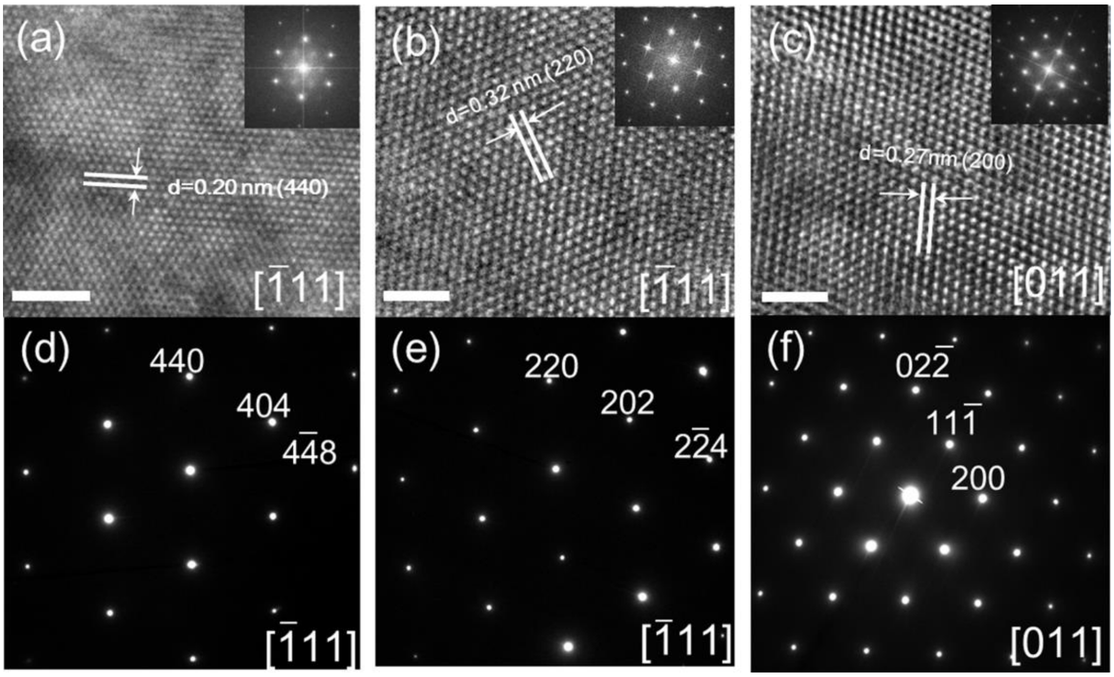

Supplementary Figure 9. HRTEM images and corresponding SAED patterns of GFCCO (a, d), CFO (b, e), and CGO (c, f) taken from the sintered CGO-CFO5050 sample. Scale bar is 2 nm.

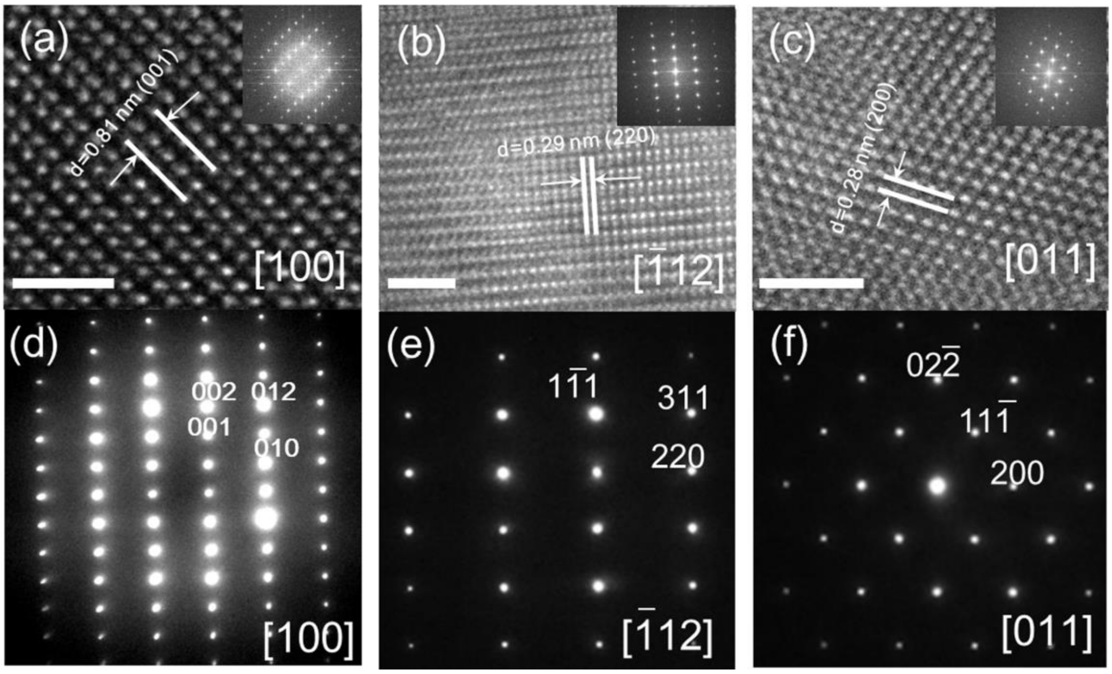

Supplementary Figure 10. HRTEM images and corresponding SAED patterns of GFCCO (a, d), CFO (b, e), and CGO (c, f) taken from the sintered CGO-CFO6040 sample. Scale bar is 2 nm.

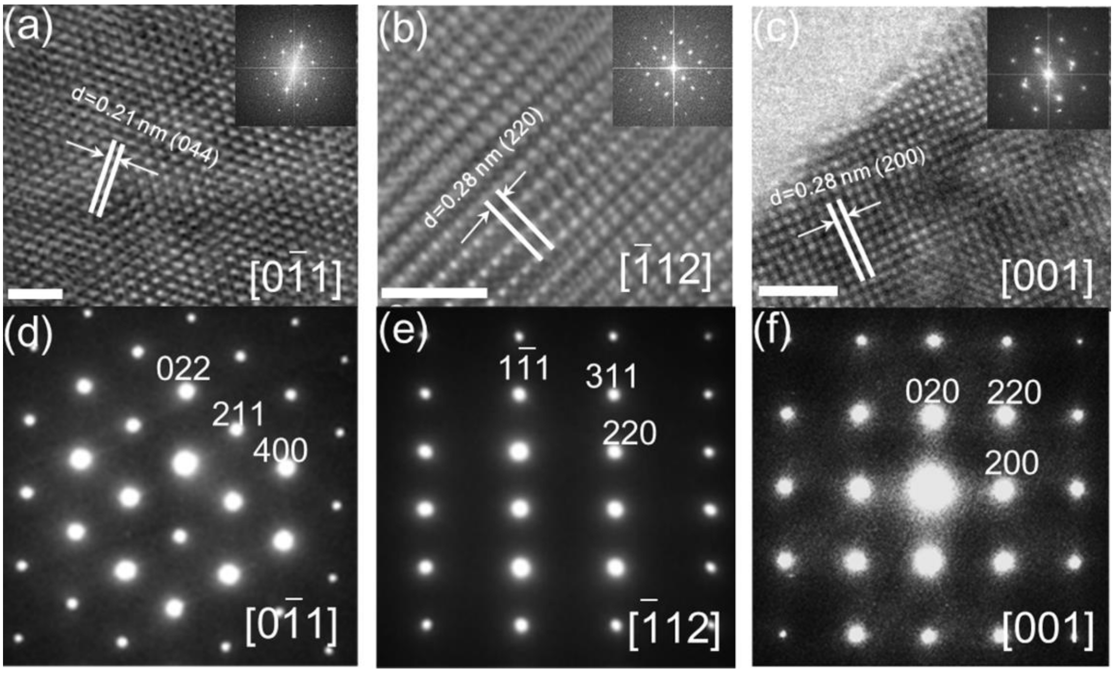

Supplementary Figure 11. HRTEM images and corresponding SAED patterns of GFCCO (a, d), CFO (b, e), and CGO (c, f) taken from the sintered CGO-CFO8020 sample. The SAED of CGO (f) showed obvious diffraction scattering effect, indicating the formation of nano domains related to the defects dissociation/association[2]. Scale bar is 2 nm.

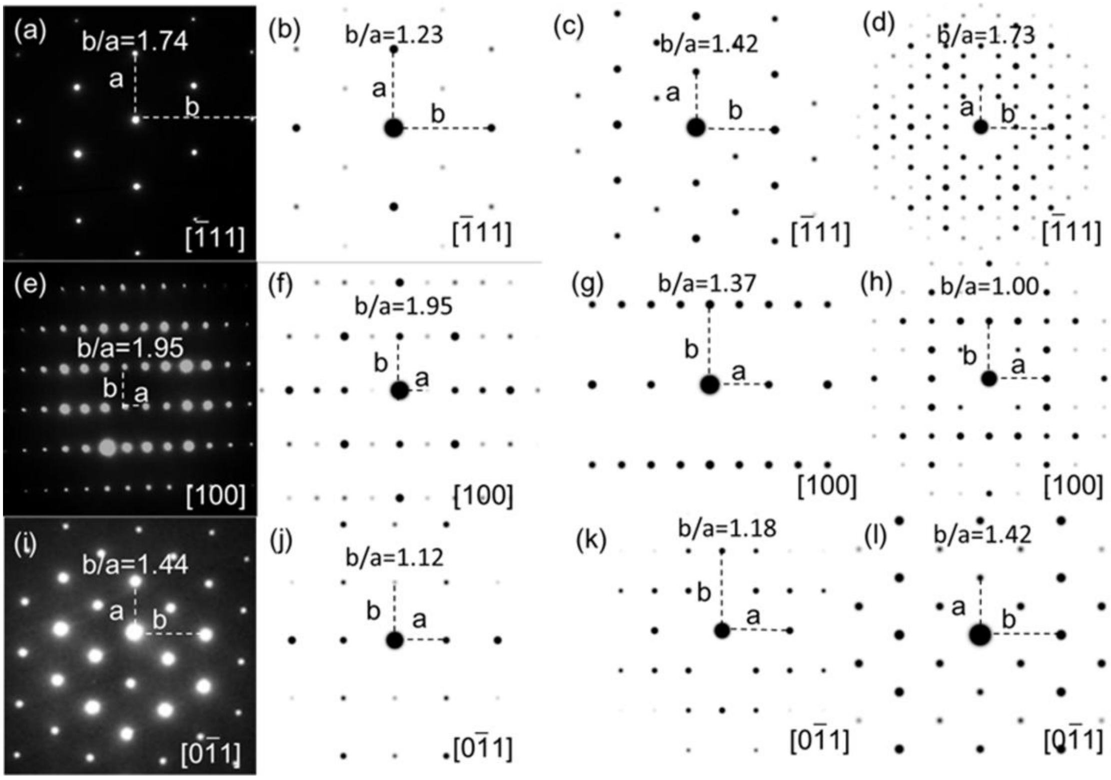

Supplementary Figure 12. **SAED patterns of GFCCO phases in the CGO-CFO composites.** (a) SAED of GFCCO grains in CGO-CFO5050 tilted in the [111] direction. (b-d) simulated diffraction patterns of $A_2B_2O_{6-\delta}$ type phase ($PrBaCo_2O_{5.51}$, ICSD-155459), ABO_3 type phase ($GdFeO_3$, ICSD-150359), and $A_3B_5O_{12-\delta}$ type phase ($Gd_3Fe_5O_{12}$, ICSD-27127) in the same zone axis. (e-h) and (i-l) are similar results for the CGO-CFO6040 and CGO-CFO8020 composites, respectively.

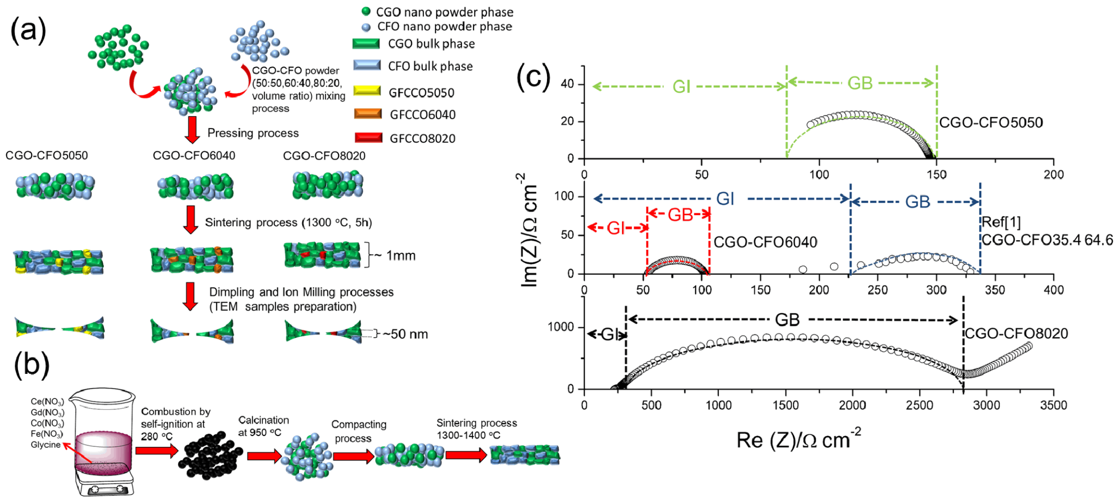

Supplementary Figure 13. (a) Fabrication process of CGO-CFO composites by powder mixing method, (b) Fabrication process of CGO-CFO composites by one pot method, (c) The impedance spectra of the CGO-CFO composites at 300 ^{o}C in air. For comparison, the results of CGO-CFO prepared by one pot method from Takamura et al. are also shown[1]. It should be mentioned that the total electrical conductivity of CGO-CFO with volume ratio of 35.4:64.6 shown in this figure is several times higher than CGO-CFO with volume ratio of 83.3:16.7 and 68.6:31.4 prepared by the same one pot method at 300 ^{o}C in air. The total electrical conductivity of CGO-CFO6040 and CGO-CFO5050 prepared by powder mixing method is much higher than those prepared by one pot method (see Supplementary Table 4 for more details).

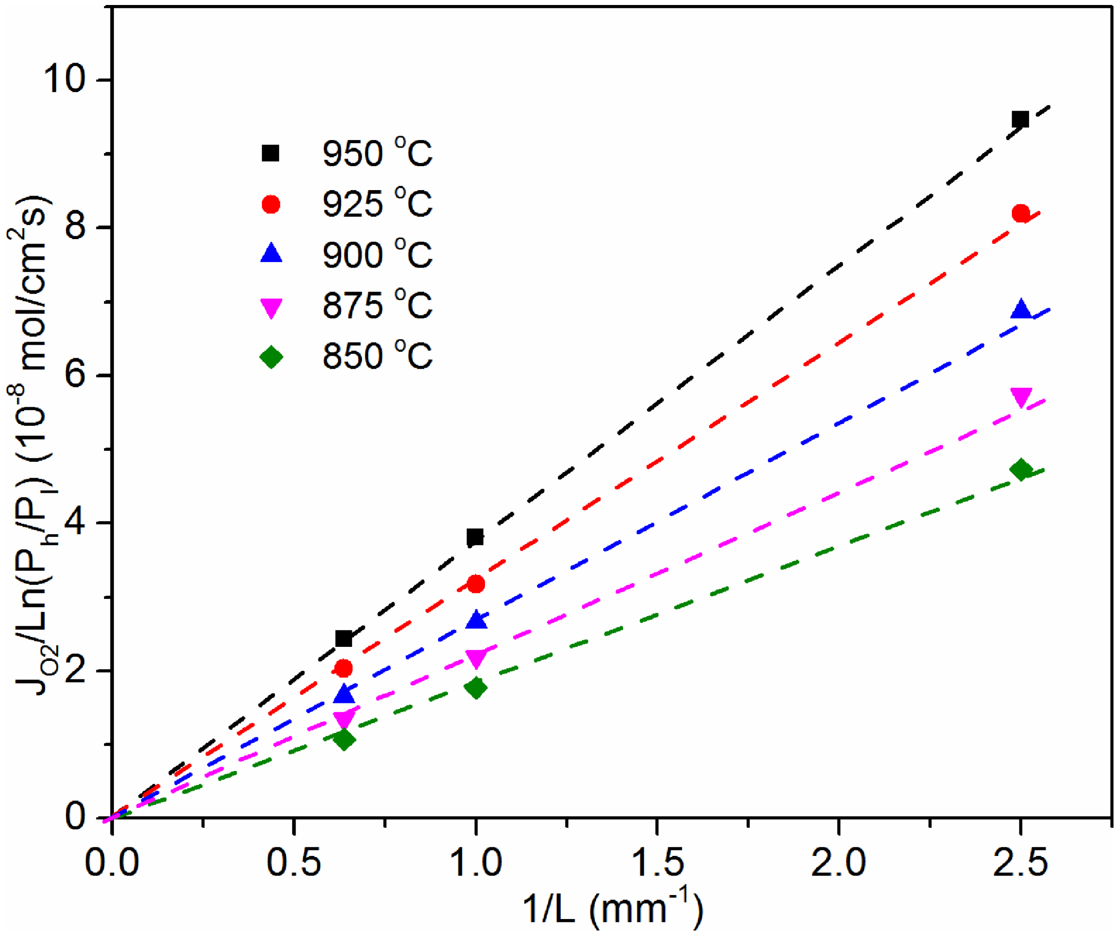

Supplementary Figure 14. Relationship between oxygen permeation fluxes and the reciprocal thickness of CGO-CFO6040 membrane at different temperatures. Membrane thicknesses are 1.57, 1.00, and 0.40 mm, respectively. Feed gas: 150 mL/min air; Sweep gas: 60 mL/min He. Both sides of the membranes were coated with CGO-SSC catalyst.

Supplementary Table 1. Atomic element ratios (%), relative atomic ratios, and averaged Z values (Z_{avg}) from different grains for the CGO-CFO5050 composite as obtained from STEM/EDX. $Z_{avg,\ GFCCO}$ is calculated in oxygen stoichiometric condition.

	**Gd**	**Ce**	**Co**	**Fe**	**Fe/Co**	**Gd/Ce**	**Gd/Fe**	**Ce+Co**	**(Gd+Ce)/(Co+Fe)**	**Z_{avg}**
**GFCCO**	40.80	4.60	6.60	48.0	7.30	9.10	0.85	11.20	0.83	22.2
**CFO**	-	-	34.3	65.7	1.92	-	-	-		16.0
**CGO**	12.40	87.60	-	-		0.14				25.3

Supplementary Table 2. Atomic element ratios (%), relative atomic ratios, and averaged Z values (Z_{avg}) from different grains for the CGO-CFO6040 composite as obtained from STEM/EDX. $Z_{avg,\ GFCCO}$ is calculated in oxygen stoichiometric condition.

	Gd	Ce	Co	Fe	Fe/Co	Gd/Ce	Gd/Fe	Ce+Co	(Gd+Ce)/(Co+Fe)	Z_{avg}
**GFCCO**	37.40	7.90	7.70	47.0	6.11	4.82	0.80	15.6	0.83	22.1
**CFO**	-	-	34.5	65.5	1.90					16.0
**CGO**	15.70	84.30	-	-		0.19				25.4

Supplementary Table 3. Atomic element ratios (%), relative atomic ratios, and averaged Z values (Z_{avg}) from different grains for the CGO-CFO8020 composite as obtained from STEM/EDX. $Z_{avg,\ \mathbf{GFCCO}}$ is calculated in oxygen stoichiometric condition.

	**Gd**	**Ce**	**Co**	**Fe**	**Fe/Co**	**Gd/Ce**	**Gd/Fe**	**Ce+Co**	**(Gd+Ce)/(Co+Fe)**	$\mathbf{Z_{avg}}$
**GFCCO**	45.00	7.00	6.40	41.6	6.48	6.49	1.08	13.40	1.08	23.1
**CFO**	-	-	44.1	55.9	1.29	-	-	-		16.1
**CGO**	9.40	90.60	-	-		0.097				25.1

Supplementary Table 4. Comparison of the grain interior(GI) and grain boundary (GB) conductivity for the CGO-xvol%CFO composites prepared using one pot method[1] and powder mixing method (this work) at 300 °C in air. The maximum GI, GB and total conductivity are found in CGO-CFO6040 and CGO-64.6%CFO composites prepared by powder mixing and one pot method, respectively. The GI, GB, and total conductivities of CGO-CFO6040 composite prepared by powder mixing method (this work) are 4.02, 2.34 and 3.18 times as high as those of CGO-64.6%CFO prepared by one pot method.

	Ref[1] x=16.7	Ref[1] x=31.4	Ref[1] x=64.6	This work x=20	This work x=40	This work x=50	CGO-CFO6040 (this work) / CGO-64.6%CFO (ref[1])
GI ($S\ cm^{-1}$)	8.44E-5	1.46E-4	4.20E-4	3.43E-4	0.00169	0.00128	4.02
GB ($S\ cm^{-1}$)	1.13E-5	9.96E-5	7.31E-4	3.48E-5	0.00171	0.00145	2.34
Total ($S\ cm^{-1}$)	9.75E-6	5.93E-5	2.67E-4	3.16E-5	8.49E-4	6.77E-4	3.18

Supplementary Note 1

Z_{avg} of different phases in all the composites is calculated based on the quantification results (Supplementary Table1-3). The average atomic number (Z_{avg}) of an oxide phase with ideal oxygen stoichiometry as $A_xB_yO_z$ was calculated using the following equation: $$Z_{avg} = \frac{(xZ_A + yZ_B + zZ_O)}{x+y+z} \quad (1)$$

where x, y, and z represent the atomic element ratio of the A, B cations and oxygen. Z_A, Z_B, and Z_O are the atomic number of the cations and oxygen. Specifically, the atomic element ratio was based on Table 1-3 and the atomic numbers were 58, 64, 27, 26, and 8 for Ce, Gd, Co, Fe and O. If there is no oxygen non-stoichiometry, the ideal z will be 1.5 in both perovskite (ABO_3) and garnet ($A_3B_5O_{12}$) when x+y=1 (Supplementary Table 1, 2, and 3). The calculated Z_{avg} of GFCCO shown in these tables were obtained under this assumption, and are lower than those of their corresponding CGO phases, which is apparently not the case considering their higher Z-contrast than those of CGO. The only explanation is that there is actually a certain oxygen non-stoichiometry (δ), which can significantly affect their Z_{avg} because oxygen has a much smaller atomic number than those of the main elements Gd, Ce, Co, and Fe. Therefore, a high level of oxygen deficiency should exist in all the GFCCO phases in order to show higher Z-contrast than the CGO phase. The actual oxygen content (1.5-δ) of GFCCO phases was obtained based on the following equation: $$Z_{avg,CGO} = \frac{xZ_A + yZ_B + (1.5-\delta)Z_O}{x+y+1.5-\delta} \quad (2)$$

Then the least oxygen vacancy concentration (c) was calculated by this equation:

$$\mathrm{c} = \frac{\delta}{1.5} \quad (3)$$

For GFCCO5050, 6040, and 8020, the calculated c is ~30%, ~32%, and ~20%, respectively. Please note these values are the least oxygen vacancy concentration in GFCCO phases to make their Z-contrast higher than those of CGO.

Supplementary Note 2

$$\sigma_{amb} = \frac{\sigma_{el}\sigma_{ion}}{\sigma_{el}+\sigma_{ion}} \quad (4)$$

$$j_{O_2} = -\frac{RT}{16F^2L}\int_{lnP_{O_2}^{'}}^{lnP_{O_2}^{''}} \sigma_{amb} dlnP_{O_2} \quad (5)$$

where σ_{amb}, σ_{ion}, σ_{el} , j_{O_2} , R, T, F, L, *and* P_{O_2} are ambipolar conductivity, ionic conductivity, electronic conductivity, oxygen flux, gas constant, temperature, Faraday constant, membrane thickness, and oxygen partial pressure, respectively.

The prerequisite for the use of the Wagner equation is that the oxygen permeation process is bulk controlled. In order to verify this, we measured the oxygen permeation fluxes of CGO-CFO6040 membrane with thickness of 1.57, 1.00, 0.40 mm (shown in Supplementary Figure 14). The fluxes is proportional to the reciprocal of thickness in the whole measurement range, suggesting that the oxygen permeation process is bulk controlled.

Supplementary References

1. Takamura, H., Kawai, M., Okumura, K., Kamegawa, A. & Okada, M. Preparation and oxygen permeability of Gd-doped ceria and spinel-type ferrite composites. *Mat. Res. Soc. Symp. Proc.* **756**, EE8.11.11-16, (2002).

2. Li, Z.-P., Mori, T., Auchterlonie, G. J., Zou, J. & Drennan, J. Direct evidence of dopant segregation in Gd-doped ceria. *Appl. Phys. Lett.* **98**, 093104, (2011).
